# A Case of Tubulointerstitial Nephritis and Uveitis (TINU) Syndrome with High ASLO Titer

**DOI:** 10.4137/ccrep.s2299

**Published:** 2009-04-09

**Authors:** Yasuyo Kashiwagi, Shunsuke Suzuki, Tao Fujioka, Shingo Oana, Hisashi Kawashima, Kouji Takekuma, Akinori Hoshika

**Affiliations:** Department of Pediatrics, Tokyo Medical University, Tokyo, Japan.

**Keywords:** TINU syndrome, ASLO titer, Lymphocyte phenotype

## Abstract

Tubulointerstitial nephritis and uveitis (TINU) syndrome is a rare autoimmune disease and the pathogenesis is still unknown. We report a case of TINU syndrome with high ASLO titer.

Uveitis improved and urine β_2_-MG normalized with low dose systemic predonisolone and cyclosporin A. The high ASLO titer in early phase suggested that streptococcal infection might have triggered TINU syndrome. Lymphocyte phenotypes normalized after treatment with low dose systemic predonisolone and cyclosporin A.

## Introduction

Tubulointerstitial nephritis and uveitis (TINU) syndrome is an uncommon subset of acute tubulointerstitial nephritis (TIN), clinically characterized by TIN with an acute favorable course and uveitis with a chronic relapsing course. Since the first description by Dobrin et al. in 1975,^1^ about 180 patients, mainly female adolescents, have been reported in nephrology and ophthalmology literatures. Drugs and infections were risk factors, but approximately half of them were idiopathic. We report here a case of idiopathic TINU syndrome with high serum antistreptolysin O (ASLO) titer.

## Case Report

A 12-year-old female was referred to our pediatric department because of high level of urinary β_2_-microglobulin (β_2_-MG).

She had ocular pain, redness and photophobia in both eyes 5 months to her hospital visit. Bilateral anterior uveitis was diagnosed by slit lamp examination; predonisolone eye drops were given. Her vital signs were within normal limit, and her physical examination was non-specific. Laboratory findings were normal except of erythrocyte sedimentation rate (25 mm/hr), ASO titer (2667 IU/ml) (normal 0–200), urinary β_2_-MG (1996 μg/l) (normal 0–200) and NAG (12.6 U/l) (normal 0–7). BUN and serum creatinine were 11.4 and 0.51 mg/dl, respectively. Antinuclear antibody (ANA), antineutrophil cytoplasmic antibody (ANCA), antidouble-stranded DNA, anti-RNP antibody, anti-Sm antibody, anti-SSA/SSB antibody, angiotensin-converting enzyme (ACE) were all negative.

Urinalysis showed microscopic hematuria and mild proteinuria (0.5 g/day). Urinary sediment contained 10–30 red blood cells/high power field (hpf) with red blood cell casts.

Lymphocyte phenotypes were as follows: CD3+ T lymphocyte 49% (normal 58%–84%), CD4+ T lymphocyte 22.1% (normal 25%–54%) and CD8+ T lymphocyte 39.9% (normal 23%–56%).

**Figure 1:**
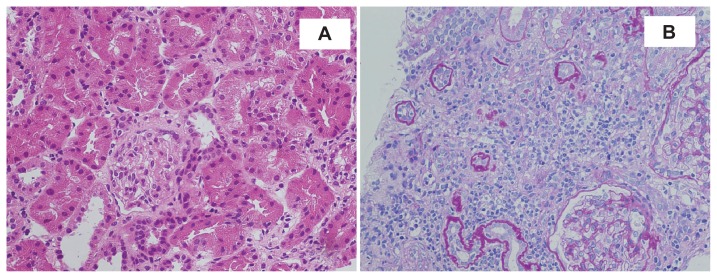
The renal biopsy shows edematous interstitium which was infiltrated with lymphocytes and plasma cells. **A**) Hematoxylin-eosin staining X200. **B**) Periodic acid schiff staining X200.

A percutaneous renal biopsy was performed immediately after admission. Light microscopic examination showed that the most characteristic finding was acute tubulointerstitial nephritis. The edematous interstitium was infiltrated with lymphocytes and plasma cells (Fig. 1). No significant increase in the extracellular matrix and number of the mesangial cells was observed in any of the glomeruli examined with normal blood vessels. No deposit was revealed by immunofluorescence or electron-microscopic studies in the glomeruli. Based on the ocular and renal findings, TINU syndrome was diagnosed.

**Figure 2:**
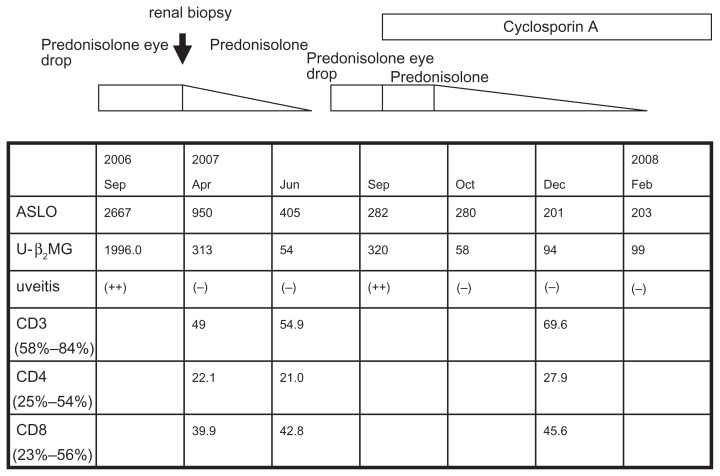
Serial change of ASLO, urine β_2_-microblobulin (U-β_2_ MG), uveitis and lymphocyte phenotypes according to clinical course and treatment.

Uveitis was not responsive to predonisolone eye drops. She was treated with systemic corticosteroids at an initial dose of predonisolone 60 mg/day (1 mg/kg/day), which was gradually tapered (Fig. 2). Uveitis improved temporarily and serum and urinary abnormalities were normalized within about 3 months. The duration of predonisolone treatment was shortened because of an increase in intraocular pressure. Lymphocyte phenotypes were not normalized after systemic predonisolone. (Jun 2007. Fig. 2) Two months later while tapering oral predonisolone, anterior uveitis recurred and urine β_2_-MG increased again. Uveitis was not responsive to predonisolone eye drops. However uveitis improved and urine β_2_-MG normalized with low dose systemic predonisolone and cyclosporin A (3 mg/kg/day) (Fig. 2). Lymphocyte phenotypes normalized after the treatment with cyclosporine (CD3+ T lymphocyte 69.6%, CD4+ T lymphocyte 27.9% and CD8+ T lymphocyte 45.6%) (Dec 2007). Predonisolone was tapered slowly over 3 months, and the uveitis is presently controlled with cyclosporine A.

## Dicussion

The pathogenesis of TINU syndrome has not been elucidated. However, infection-induced cases were published by Ljutic and Glavina^2^ (varicella zoster), Cigni et al.^3^ (Epstein-Barr virus), Deguchi and Amemiya^4^ (HTLV-1). In our case, her ASLO titer was up to 2667 IU/ml (Sep 2006) and it was decreased to 282 IU/ml by 12 months (Sep 2007). The high ASLO titer in the early phase suggested that streptococcal infection might have triggered TINU syndrome. There are few cases of TINU syndrome with high ASLO titer. Koike et al.^5^ reported a case of TINU syndrome with full type Fanconi syndrome and high ASLO titer. Two cases, including our case, were reported by in Japanese.

Mandeville JH et al. have reported the human leukocyte antigen (HLA) specificities of patients with TINU syndrome.^6^ The most commonly reported HLA specificities have been HLA-A2 and HLA-A24 (a serologically defined subgroup of HLA-A9). In particular, HLA-A24 (or -A9) was identified in 75% of Japanese patients. Both specificities are common in Asian patients, however, and thus they may not be related to the disease. (HLA-A24 is present in 9.5%–61.0% of Asian patients. HLA of our case was not determined).

The relationship between the type of HLA and the pathogenesis of TINU or streptococcal infection remains poorly understood.

In our case, peripheral CD3+ T lymphocyte and CD4+ T lymphocyte were decreased even after systemic steroid therapy although uveitis recovered temporarily and urine β_2_-MG normalized. Lymphocyte phenotypes normalized after the treatment with cyclosporine. Jung WL et al. reported a case of idiopathic TINU syndrome with severe immunologicdysregulation.^7^ Alymphocyte-mediated immune mechanism has been strongly suggested for a pathogenesis of idiopathic TINU.^6^,^8^ Yoshioka et al.^9^ demonstrated renal infiltration by activated T cells with IL-2 receptors, Takemura et al.^10^ reported increased peripheral B lymphocytes, T lymphocytes and macrophages in the renal interstitium in the early acute phase. Gafter et al.^11^ proposed suppressed peripheral cellular immunity, associated with active local immunity by demonstrating a reduction in peripheral T lymphocytes (T helper cell), decreased lymphokine secretion and the emergence of T lymphocytes at renal inflammatory sites. An analysis of a lymphocyte-mediated immune mechanism is still controversial. In our case we didn’t have the data on the subset of lymphocytes infiltrated in renal interstitium. The renal disease in TINU syndrome seems to have an excellent prognosis, with complete resolution with or without corticosteroid; 80% received systemic corticosteroids with rare relapse, although in a small percentage of adult, mild residual renal insufficiency was seen.^12^

Uveitis in the TINU syndrome responds less promptly to corticosteroids and tends to relapse.^10^ In this case, uveitis was not completely responsive to high dose systemic predonisolone, but improved with cyclosporine A and lymphocyte phenotypes normalized after cyclosporine A.

The exact etiology of TINU syndrome is still unknown. Long term follow-ups for immunologic studies and uveitis are required.

## References

[1] Dobrin RS, Vernier RL, Fish AJ (1975). Acute eosinophilic interstitial nephritis and renal failure with bone marrow-lymph node granulomas and anterior uveitis. Am J Med.

[2] Ljutic D, Glavina M (1995). Tubulointerstitial nephritis with uveitis syndrome following varicella zoster reactivation. Nephron.

[3] Cigni A, Soro G, Faedda R (2003). A case of adult-onset tubulointerstitial nephritis and uveitis (TINU syndrome) associated with sacroileitis and Epstein-Barr virus infection with good spontaneous outcome. Am J Kidney Dis.

[4] Deguchi HE, Amemiya T (2003). Two cases of uveitis with tubulointerstitial nephritis in HTLV-1 carriers. Jpn J Ophthalmol.

[5] Koike K, Usui M, Matsumoto Y (2007). Adult-onset acute tubulointerstitial nephritis and uveitis with Fanconi syndrome. Clin Nephrol.

[6] Mandeville JH, Levinson RD, Holland GN (2001). The tubulointerstitial nephritis and uveitis syndrome. Surv Ophthalmol.

[7] Jung WL, Hyun JK, Soon HS, Seung JL (2005). A case of tubulointerstitial nephritis and uveitis syndrome with severe immunologic dysregulation. Pediatr Nephrol.

[8] Vohra S, Eddy A, Levin AV, Taylor G, Laxer RM (1999). Tubulointerstitial nephritis and uveitis in children and adolescents. Four new cases and a review of the literature. Pediatr Nephrol.

[9] Yoshioka K, Takemura T, Kanasaki M, Akano N, Maki S (1991). Acute interstitial nephritis and uveitis syndrome: activated immune cell infiltration in the kidney. Pediatr N.

[10] Takemura T, Okada M, Hino S (1999). Course and outcome of tubulointerstitial nephritis and uveitis syndrome. Am J Kidney Dis.

[11] Gafter U, Kalechman Y, Zevin D (1993). Tubulointerstitial nephritis and uveitis: Association with suppressed cellular immunity. Nephrol Dial Transplant.

[12] Burnier M, Jaeger P, Campiche M (1986). Idiopathic acute interstitial nephritis and uveitis in the adult. Report of 1 case and review of the literature. Am J Nephrol.

